# Responses of Ecosystem Services to Urbanization-Induced Land Use Changes in Ecologically Sensitive Suburban Areas in Hangzhou, China

**DOI:** 10.3390/ijerph16071124

**Published:** 2019-03-28

**Authors:** Shaofeng Yuan, Congmou Zhu, Lixia Yang, Fenghua Xie

**Affiliations:** 1 School of Public Administration, Zhejiang Gongshang University, Hangzhou 310018, China; shaofengyuan1975@163.com; 2 Institute of Applied Remote Sensing and Information Technology, College of Environmental and Resource Sciences, Zhejiang University, Hangzhou 310058, China; congmouzhu1993@163.com; 3 School of Public Administration, Zhejiang University of Finance & Economics, Hangzhou 310018, China; yanglixianj2001@163.com; 4 School of Business Administration, Zhejiang University of Finance & Economics, Hangzhou 310018, China

**Keywords:** ecosystem services value, land use, temporal–spatial change, ecologically sensitive suburban area, Hangzhou

## Abstract

Ecologically sensitive suburban areas provide important ecosystem services and protect urban ecological security because of their multiple functions in natural and human systems. The research on the ecological environment effects of land use activities in ecologically sensitive suburban areas is important in guiding the healthy and sustainable development of cities. Taking the west suburbs of Hangzhou in China as a case study, we quantified land use changes from Landsat satellite imagery and calculated the value of ecosystem services using the well-established equivalent factor table for land use/cover change (LUCC) and ecosystem services value (ESV). The impacts of LUCC on the ecological environment were analyzed using the transfer matrix of land use and coefficient of elasticity. Results revealed the following. (1) The total ESV in the western suburban area of Hangzhou decreased from $109.95 million in 2000 to $87.09 million in 2016. Moreover, the ESV of gas regulation, climate regulation, soil formation and protection, as well as biodiversity conservation presented a large decrease of more than 25%, especially between 2010 and 2016. (2) The spatial distribution of ESV was high in the west and low in the east. The regions with a significant reduction in ESV were mainly distributed in the eastern town of Wuchang and in Jincheng Town located in the midwest valley. (3) Industrial agglomeration activities in the ecologically sensitive suburban area emerged as the primary factor influencing ESV for various land uses. The elasticity indicator for assessing the responses to ESV changes relative to LUCC showed that 1% of the land conversion in this area resulted in average changes in ESV of 4.1% after the establishment of the industrial agglomeration area. (4) The increase in construction land was associated with a significant decrease in forest area because of the policy of cultivated land requisition–compensation balance and development strategies for low-slope hilly lands. Consequently, the ESV in the ecologically sensitive suburban areas rapidly declined.

## 1. Introduction

Ecosystem services are defined as the benefits that humans can obtain directly or indirectly from natural ecosystems [1,2]. Ecosystems not only provide a wide variety of materials (such as food, water, soil, and other raw materials) but also offer non-material services (such as gas regulation, climate regulation, and aesthetic benefits) that are essential to sustaining the daily lives of organisms on Earth [3,4]. Since the end of World War II, the world’s population has become increasingly concentrated in urban areas [5]. The growth and expansion of metropolitan regions where agricultural and non-agricultural activities are spatially integrated make the distinction between rural and urban land uses especially problematic [6,7,8]. Suburban areas, which refer to important ecological areas, include valuable protected biotopes, such as forests, preserved grasslands, high-quality agricultural lands, and important wetlands; and they often provide urban residents with essential ecosystem services that are not available elsewhere due to the special geographical locations of these areas [9,10,11]. However, the degree of systematic preservation and protection of ecosystems in suburban areas is not commensurate with their importance; uncontrolled land development and over-exploitation of suburban resources have massively altered natural and semi-natural environments and have led to a great reduction in the ecological environmental capacity of suburban areas [12]. The suburban urbanization in megacities has been aggressive in China because of the growing number of science and technology parks and economic and industrial parks built in suburban areas, and an increasing conflict between urban expansion and ecosystem protection has been identified in the literature [10,13,14]. Hence, an estimation of the monetary value of the ecosystem services of suburban areas could help to provide environmental managers with a realistic indication of the ability of suburban ecosystems to support current land use practices. It could also build a solid foundation for the rational and sustainable utilization of environmental resources.

Ecosystem services were first studied in the 1970s, and they have gradually developed as a research focus in the last 50 years. One of the earliest and most influential pioneers of this research is Costanza, who created a methodological framework that employs a range of market, non-market, and biophysical valuation methods to estimate global ecosystem services value (ESV) from 17 types of service functions; this framework has been widely adopted and improved by subsequent researchers [15,16]. Over the years, research projects on global and regional ecosystem services led by national and international organizations have gradually increased in number, thereby facilitating scientific decision making for global ecological and environmental management [14,17,18]. On the basis of the research findings of Costanza and local ecological characteristics, Xie et al. improved Costanza’s approach by combining the views of 200 domestic scholars about ecosystem services in China to modify their estimates of global ESV for China [19,20]. Xie et al.’s method is considered practical in China and has thus been widely used by other scholars in the country [21].

For the evaluation of ESV, a group of three approaches are available in the literature: Willingness to pay (WTP) methods, ecological process–benefit assessments, and land use/cover change (LUCC)-based methods [11,22]. Of these approaches, the evaluation methods based on LUCC have been popularly applied to research due to the rapid development of geographic information systems and remote sensing technologies [12,23]. A large number of studies on the evaluation of ESV have been undertaken in relation to LUCC on global [24], national [19], regional, and basin scales [3]. Generally, urbanization is proved to have a negative impact on ecosystem services. Costanza et al. revealed that global land use changes between 1997 and 2011 caused a loss in ESV of US$ 20.2 trillion/year [16]. Previous studies on the impacts of urbanization in China also confirmed the findings. For example, Wu et al. found a total decrease of 24% (US$ 111.91 million) under urbanization in Hangzhou from 1978 to 2008 [25]. Peng et al. revealed that the impact of urban sprawl on ecosystem services is more direct and effective than that of population growth and economic development [11]. Scholars possess a well-developed understanding of the negative impact of urbanization on ecosystem services. However, most existing studies focused on global or regional land use changes and the quantitative consequences of ecosystem service loss, while only a few studies analyzed the urban suburbs in China that are undergoing drastic land use changes in recent years. In addition, the factors that could affect ecosystem service changes in suburban regions have yet to be specified clearly.

Located in the wealthy eastern region of China, the suburbs of Hangzhou have experienced rapid urbanization and drastic land use changes since the reform and opening up in the 1980s. Additionally, the western suburban area of Hangzhou is rich in forest and cropland and presents excellent eco-environmental quality. In 2010, the agglomeration of the science and technology innovation industry was established in the western suburban area of Hangzhou so that the comprehensive functions of the suburbs in Hangzhou could be fully utilized to support urban economic transformation and development. However, the ecological impacts of suburban land use changes have not been analyzed despite such analysis being essential for urban sustainable development. The western suburban area in Hangzhou thus serves as an excellent case study to explore the ecological impacts of land use changes in metropolitan suburbs and could provide a reference for urban land use management and sustainable development in this region.

The present study aims to examine land use changes and their impacts on ecosystem services in response to rapid urbanization in metropolitan suburbs on the basis of the case of Hangzhou city. The specific objective of this work is to assess the dynamic changes in ESV and evaluate the impacts of industrial agglomeration activities on ecosystem services in the western suburban area of Hangzhou. The remainder of this paper is organized as follows. Section 2 introduces the study area and the research methods. Section 3 presents the land use changes and their impacts on ecosystem services. Section 4 provides a discussion in relation to existing references. Section 5 concludes the study.

## 2. Materials and Methods

### 2.1. Study Area

**Figure 1:**
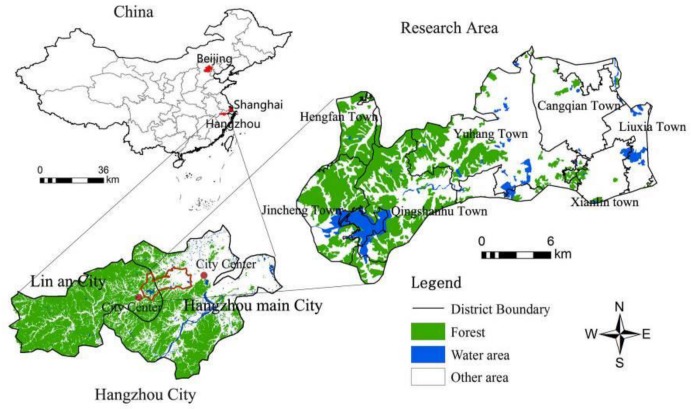
Location and administrative division of the ecologically sensitive suburban areas in Hangzhou.

The western suburbs of Hangzhou represent one of the most important ecologically sensitive areas in the Middle-Lower Yangtze Plain. The agglomeration of the science and technology innovation industry was established in the ecologically sensitive area of the western suburbs of Hangzhou in 2010. This area is related to the northwestern and southwestern ecological belts and belongs to the key area of ecological protection. The total planning area was about 300 km^2^, and the planning scope involved 10 major towns. Figure 1 shows the location, topography, and administrative divisions. This area includes the Yuhang Innovation Base and Qingshanhu Science and Technology Town. The characteristics of the geographical environment in this region are as follows: The terrain is high in the west and low in the east; the ecological lands such as forests, grasslands, and lakes are distributed in the west; and the cultivated land and wetland are located in the east.

After the establishment of the industrial agglomeration, a large number of high-end enterprises and talents settled in this area. In 2015, the GDP in this area was about 50 billion yuan, which was twice that in 2009. Moreover, over 480 enterprises of standard size settled in the industry agglomeration in western Hangzhou. The rapid agglomeration of industries leads to dramatic changes in land use patterns and causes ecological pressure and land use spatial conflicts.

### 2.2. Data Sources

The main data source of the study was the Landsat-5 TM remote sensing images with seven spectra and a ground resolution of 30 m. The TM remote sensing images of Hangzhou in 2000, 2005, 2010, and 2016 were selected for the study. The remote sensing interpretation data of 2000, 2005, and 2010 were provided by the Resources and Environmental Sciences Data Center, Chinese Academy of Sciences, which had been widely used in researches on land use changes [7,8]. These land use datasets include information on land use categories, namely, forest, grassland, cultivated land, water area, urban construction land, rural settlements, and unused land. The land use data in 2016 were generated by Landsat 8 Operational Land Imager images. High-resolution remote sensing data were then used to further modify the remote sensing interpretation data to improve accuracy. Socio-economic data were adopted from the Hangzhou Statistical Yearbook for 2000–2016. The other data were mainly from the conceptual planning for the agglomeration of the science and technology innovation industry in Hangzhou (2013).

### 2.3. Estimation of ESV

In this study, we primarily followed the research results of Costanza et al. and Xie et al. to calculate the ESV in the suburban area under study [19,26]. First, we divided the western suburban area of Hangzhou into nine types of services in five ecosystems and then determined the equivalent coefficients of the ESV for all land use types (Table 1). However, Xie et al.’s value coefficients were established at the national scale, and significant divergence can occur at the local level [27,28]. Hence, we tailored the value coefficients to local conditions by reconsidering the ESV of construction land. In the western suburb area of Hangzhou, the built-up area includes green spaces such as parks, scenic spots, and residential greenland; and the surrounding environment of rural settlements is better than that of urban villages [6,13]. Thus, we determined the equivalent coefficient of the ESV for construction land on the basis of the study results by Chuai et al. [29]. 

Second, we used Xie et al.’s definition in setting the equivalent coefficient of the ESV per unit area to be equal to the value of natural food production per hectare per year, i.e., one-seventh of the value of actual food production [30,31]. In Hangzhou, the annual average output value of crops between 2000 and 2015 was 284.99 US$.hm^−1^ a^−1^. Based on Xie et al.’s results, the final equivalent coefficients of the per-unit ESV for different land use types are shown in Table 2.

Third, on the basis of Table 2 and the assessment model proposed by Barbier et al. [32], the total ESV was calculated through the sum of all ESV obtained by multiplying the area of each land use type by the value coefficient of the corresponding ecosystem (Equation (1)).

(1) $$ESV_{j}=\sum_{i=1}^{n}L_{i}\times VC_{ij}$$

(2) $$ESV=\sum_{i=1}^{n}L_{i}\sum_{j=1}^{m}VC_{ij}$$

where *ESV_j_* is the estimated ESV of function *j* in the study area, *VC_ij_* is the value per unit area of ecosystem service *j* of land use type *i*, *L_i_* is the area of land use type *i*, *ESV* is the total ESV in the study area, *n* is the number of land use types, and *m* is the number of ecosystem service types.

### 2.4. Elasticity of ESV Change in Relation to LUCC

Elasticity is a measure of how responsive a variable is to changes in another variable. In this work, we use this method to measure percentage changes in ESV resulting from percentage changes in LUCC. Thus,

(3) $$\begin{array}{l} E=\left|\frac{(ESV_{t+1}-ESV_{t})/ESV_{t}\times 100\%}{LUP}\right|\\ LUP=\frac{\sum_{i=1}^{n}\Delta L_{i}}{\sum_{i=1}^{n}L_{i}}\times \frac{1}{T}\times 100\% \end{array}$$

where *E* is the elasticity of ESV change in relation to LUCC, *ESV_t+1_* is the ESV at the next research period, *ESV_t_* is the ESV at the start of the research period, *LUP* is the land conversion percentage, Δ*L_i_* is the converted area of land use type *i*, *L_i_* is the area of land use type *i*, and *n* is the number of land use types.

## 3. Results

### 3.1. Analysis of Land Use Dynamics

**Figure 2:**
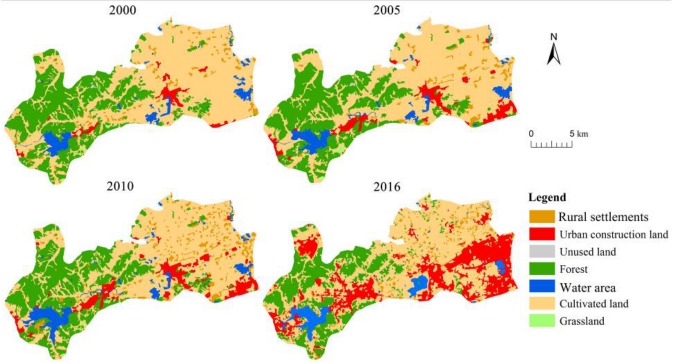
Land use pattern of ecologically sensitive area of Hangzhou in 2000–2016.

The general trends of land use changes between 2000 and 2016 in Hangzhou’s western suburban area are displayed in Table 3 and Figure 2. The area covered by cultivated land occupies the largest proportion of the total area, followed by forest, urban construction land, water area, rural construction land, grassland, and unused land. Therefore, the study area is an important ecosystem source in Hangzhou. Urban construction land underwent the most significant changes (Table 3). Its area continuously increased by nearly nine-fold from 7.67 km2 in 2000 to 65.35 km2 in 2016. This case was true especially from 2011 to 2016, with the area increasing by 43.33 km2, which accounted for 75.12% of the total increase between 2000 and 2016. By contrast, the area under cultivated and forest lands decreased by 28.93 and 30.81 km2 from 2000 to 2016, respectively, and became the main source of the expansion of urban land. Meanwhile, the areas covered by rural settlements, water area, and grassland increased by 8.61, 3.87, and 0.08 km^2^, respectively. Meanwhile, rural settlements and water area experienced a rapid decrease from 2010 to 2016.

### 3.2. Response of ESV to Land Use Changes

#### 3.2.1. Changes in ESV of Different Ecosystem Services

**Figure 3:**
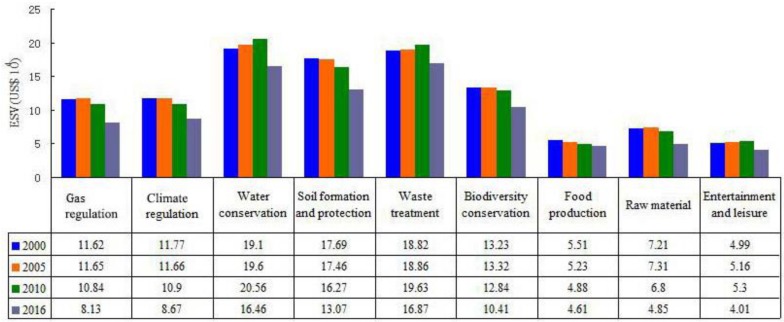
ESV in western suburban area of Hangzhou from 2000 to 2016.

The ESV of each ecosystem service type in 2000–2016 was calculated using the per-unit ESV for different land use types in Table 2, and the results are in Figure 3.

During 2000–2016, water conservation accounted for the largest proportion of the total ESV in the study area. Its value was $19.1 million in 2000 and increased to $20.56 million in 2010, but dropped to $16.46 million in 2016. The next largest proportion was that of waste treatment, whose value was $18.82 million in 2000 and increased to $19.63 million in 2010, but dropped to $16.87 million in 2016. The three lowest ESV were those for raw material, food production, and entertainment and leisure, which did not exceed $8 million. In addition, the ESV of soil formation and protection decreased the most, losing a total of $4.62 million (accounting for 20.21% of total loss) during the study period. The ESV losses of raw material, biodiversity conservation, water conservation, gas regulation, and climate regulation were relatively close, reaching approximately $2.5 million. The minimum ESV loss was for food production, which was below $1 million.

**Figure 4:**
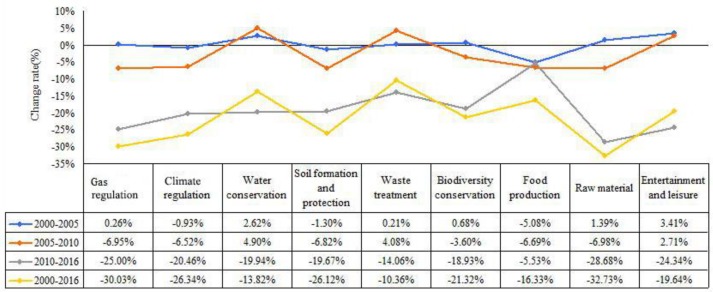
Change rate of ESV in ecologically sensitive area of Hangzhou from 2000 to 2016.

According to the results of the overall change rate of the ESV from 2000 to 2016 (Figure 4), the total ESV of raw material showed a rapid decline trend, decreasing by 32.73% during the 16 years. The ESV change rates of gas regulation, climate regulation, soil formation and protection, and biodiversity conservation presented large reductions exceeding 20%; such declines had a significant relationship with the unit values and the decreased areas of the forest and water area. Moreover, the decline rate of each ESV significantly increased after the establishment of industry agglomeration in the western suburban area of Hangzhou. Such rates became approximately 20%, except that of food production, which decreased by only 5.53%.

#### 3.2.2. Changes in ESV of Different Land Use Types

The values for the various ecosystem services were estimated with use of Equations (1) and (2), data pertaining to the seven identified land use types in Hangzhou’s ecologically sensitive area, and the unit values for the nine main ecological service types (Table 4). The total ESV of the study area in 2000, 2005, 2010, and 2016 were $109.95 million, $110.28 million, $108.04 million, and $87.09 million, respectively. These values increased from 2000 to 2010 and then rapidly decreased afterward. 

The unit service values of the ecological land (grassland, cultivated land, forest, and water area) represented in the study area were much higher than those of the covered artificial land (urban land and rural settlements). However, the degree of variation in the total ESV of the ecological land between 2000 and 2016 was considerably pronounced, representing a decrease in value of 21.47% over the 16 years. This case was especially true for the ESV of forest, which decreased by $19.18 million. Conversely, the degree of variation in the value of construction land was relatively small; its ESV increased from $0.22 million in 2000 to $0.93 million in 2016. 

#### 3.2.3. Spatial Patterns of ESV 

**Figure 5:**
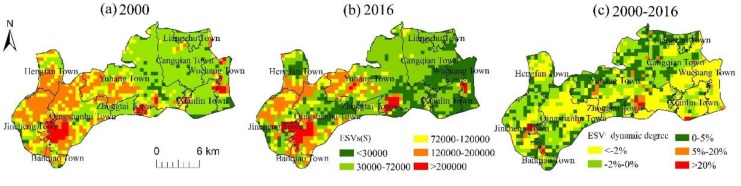
(**a**) Distribution of ESV in 2000; (**b**) Distribution of ESV in 2016; (**c**) ESV dynamic degree of ESV between 2000 and 2016.

Figure 5 shows the spatial patterns of ESV for Hangzhou’s western suburban area in 2000 and 2016 based on a 0.5 km × 0.5 km grid analysis. The spatial distribution of the region with high ESV was consistent with those of the forest and water area, which were mainly distributed in the towns of Jincheng, Qingshanhu, and Yuhang. The spatial distribution of the region with low ESV was consistent with that of the urban built-up area, and showed a trend of expansion with the urban expansion. The region with low or medium ESV was remarkably consistent with the distribution of cultivated land. All in all, the total ESV of Hangzhou’s western suburban region was noticeably high in the west and low in the east.

The average and total ESV in the different towns and their change rates were separately counted and are shown in Table 5. The ESV in all towns continuously decreased and showed a significant spatial variation. As seen in Figure 5c and Table 5, the area with an ESV reduction of more than 20% was mainly distributed in the eastern urban built-up area and the midwest valley, especially in the towns of Wuchang, Xianlin, and Hengfan, which had decline rates of 67.71%, 42.17%, and 31.49%, respectively. The region with an ESV reduction of less than 2% was mainly distributed in the transition area between the ecological and urban lands, which were located in Liangzhu Town and Yuhang Town and had negative change rates of 5.35% and 9%, respectively. 

### 3.3. Impact of Land Use Changes on ESV

To show the effects of land use changes on ESV, we created a transition matrix of the land uses and the ESV between 2000 and 2016 (Table 6). The matrix showed that the area affected by land use changes measured 130.62 km^2^, representing 42.75% of the total study area and causing a $22.88 million decrease in total ESV from 2000 to 2016. The reduction in cultivated land, which was mainly a result of urban construction expansion, accounted for 47.86% of the overall decrease in the study area and led to a US$8.53 million ESV decrease. However, the conversion from cultivated land to forest resulted in an ESV increase of US$10.65 million, which was the most significant change in conversion type that increased ESV. Meanwhile, 48.29% of the rural construction land was transformed into cultivated land, resulting in an increase of US$0.95 million. In addition, the decrease in forest, which was mainly caused by cultivated land supplementation, resulted in the largest ESV decrease of US$18.83 million, which was the most significant change in conversion type that increased ESV. The effects of the other land sue types were not as noticeable as the above mentioned ones.

To quantitatively measure the impact of land use changes on the ESV in suburban areas, we used the elasticity of ESV in relation to LUCC; that is, we measured the percentage changes in the ESV caused by percentage changes in LUCC (Table 7). The elasticities of ESV change with respect to land use changes during 2000–2005 and 2006–2010 were 0.19 and 0.84, respectively, indicating that a conversion of 1% in land use would result in average ESV changes of 0.19% and 0.84%, respectively. However, during 2011–2016, the elasticity sharply increased to 4.08, indicating that a conversion of 1% in land use would result in an average change of 4.08% in ESV. The impact of land use change on ESV was remarkably in Hangzhou’s western suburban area after 2010.

High elasticity was spatially concentrated in eastern towns. In Liangzhu and Wuchang, the average elasticity of ESV was greater than 2 during 2000–2010. During 2011–2016, the elasticity of most towns increased to more than 2, especially those of Zhongtai, Banqiao, and Hengfan. The areas whose ESV changes were most sensitive to land use changes were mainly those exhibiting low ESV. The three high-elasticity towns, mainly located in eastern plains, experienced significant decreases in ESV. Moreover, the change rate of land use was low, but ESV decreases in the western valley, such as Qingshanhu Town and Jincheng Town, were much greater than those in eastern towns. The sharp elasticity increases in these areas meant that a slight change in land use could result in significant changes in ESV.

## 4. Discussion

Dramatic land use changes are generally believed to negatively affect ecosystem services [7,33]. Suburban ecosystems could provide the necessary condition for sustainable urban development, such as forest ecosystems, which can provide gas regulation, soil formation and protection, and biodiversity conservation [34,35]. Furthermore, the forest is an ideal place where urban residents can go for leisure and holiday. Our study revealed the spatiotemporal pattern of ESV in relation to land use changes, and identified the primary land use types that cause ESV decline in the western suburban area of Hangzhou, China. Our findings agreed with those of other studies; that is, construction land expansion could lead to an ESV decrease [14,36,37,38].

Previous studies showed ESV decrease rates of 1.71% per year from 2004 to 2014 in Yinigkou City, China [17]; 0.26% per year from 1976 to 2013 in Jilin City, China [39]; and 0.89% per year from 1994 to 2014 in Hangzhou, China [40]. Our research in the western suburban area of Hangzhou indicated an ESV decrease rate of 1.30% per year during 2000–2016, which was faster than that for Hangzhou’s inner city. This result occurred because the study area we selected is rich in ecological resources, and the urban development between cities has different development strategy orientations. The following are the specific reasons for the relatively high rate of ESV loss under the negative impacts of rapid urbanization in the western suburban area of Hangzhou.

First, the establishment of the agglomeration of the scientific and technology–innovation industry in western Hangzhou in 2010 has led to a rapid increase in the reclamation of construction land from ecological and agricultural lands, thereby further expanding the urban development space. Consequently, the ecosystems in this area were damaged, given that industry agglomeration activities destroyed the original natural ecological landscape; in addition, the underlying surface properties and biodiversity were affected, thereby decreasing the ESV of the suburban industrial agglomeration area in western Hangzhou [23]. Second, utilization strategies for developing the low-slope hilly land in Zhejiang Province have been strongly supported to alleviate the contradiction between land supply and demand in recent years. The development plan of agglomeration of the scientific and technology–innovation industry in western Hangzhou encourages the use of low-slope hilly lands in the ecologically sensitive western suburban area to reduce the occupation of cultivated land in the eastern plains by local land managers. Such occupation is the reason behind the rapid decrease in the forest area located in the ecotone between agriculture and forestry after 2010. In turn, this fast decline resulted in the deterioration of the ecological environment and other economic development problems, thereby ultimately affecting urban stability and development sustainability. Third, our findings indicated that the changes in the area of cultivated land were not as noticeable as those of other land use types; this case was also true for the ESV of food production, possibly because of the strict implementation of the policy of cultivated land requisition and compensation [41,42]. Supplementary cultivated land mainly comes from forest and rural construction land in land consolidation projects (Table 6), and this condition further caused the forest area to decrease sharply from 2000 to 2016. In summary, the comprehensive effects of industry agglomeration activities and cultivated land protection policies led to the rapid decline of ESV in Hangzhou’s western suburban area.

With Hangzhou’s selection as the National Ecological Garden City, consideration for its ESV is vital for enhancing sustainable land use management in its urban areas, especially those surrounding metropolitan areas, which could provide important ecological resources for residents [13]. The absolute decrease found in the value of ecosystem services supply is an important finding, as it indicates an overall decline in the sustainability of land use and ecosystem services. Given the ongoing trends of land urbanization and socioeconomic development in the western suburban areas of Hangzhou, increasing pressure on ecological land ought to be prioritized for the sustainability of the ecosystem services in its rapidly urbanizing areas [43].

Decision-making and prioritization for the management of ecosystem services involve many complex environmental, economic, and social considerations. In the western suburban area of Hangzhou, the objectives of land use planning should be expanded to attach importance to the construction of ecological civilization while remaining cognizant of the ecosystem services that the natural forest could provide for urban sustainable development. This goal could be achieved via delineating an ecological red line and sustainability targets for the local ecosystem services. Ultimately, this knowledge will move cities in the direction of the development goal of sustainability.

## 5. Conclusions

In this work, GIS technology and land use data were used to assess changes in ESV from 2000 to 2016 and to analyze the impact of land development activities on ESV in Hangzhou’s western suburban area over this period. The main conclusions can be summarized as follows.

Land use types in Hangzhou’s western suburban area changed significantly from 2000 to 2016, especially between 2010 and 2016. The most noticeable change of these land use types was an increase of 36.38 km2 in construction land. Meanwhile, the area of cultivated and forest lands decreased by 28.93 and 30.81 km^2^, respectively, due to industry agglomeration activities. This development markedly affected the aerial distribution of land use in the area.

The total ESV in Hangzhou’s ecologically sensitive area decreased from $109.95 million to $87.09 million between 2000 and 2016 (a decline of 32.73%). Among these ecosystem services, water conservation, waste treatment, and soil formation and protection accounted for the three largest proportions of the total ESV. Over this period, raw material, gas regulation, and soil formation and protection experienced a noticeable decline, decreasing by more than 20%, especially in 2011–2016. The ESV provided by each land use type significantly differed. The change rate of the ESV of the ecological lands (such as forest and cultivated lands) was considerably more pronounced than that of the artificial lands (such as rural and construction lands).

The establishment of the agglomeration of the scientific and technology–innovation industry in western Hangzhou led to a rapid increase in construction land. Land development activities thus emerged as the main factor influencing the changes of the total ESV in Hangzhou’s sensitive suburban area. In addition, the elasticity of ESV changes with respect to land use change during 2000–2005 and 2006–2010 were lower than 1 but rapidly increased to 4.08, indicating that a conversion of 1% in land use change would result in an average ESV change of 4.08%. Finally, the impact of land development activities on ESV was remarkable in Hangzhou’s western suburban area after 2010.

## Figures and Tables

**Table 1:** Equivalent coefficients of ecosystem services value (ESV) units for different land use types in the western suburban area of Hangzhou.

Functions	Land Use Types				
	Grassland	Cultivated Land	Forest	Water Area	Construction Land
Gas regulation	0.80	0.50	3.50	0.00	0.09
Climate regulation	0.90	0.89	2.70	0.46	0.08
Water conservation	0.80	0.60	3.20	20.38	0.08
Soil formation and protection	1.95	1.46	3.90	0.01	0.09
Waste treatment	1.31	1.64	1.31	18.18	0.04
Biodiversity conservation	1.09	0.71	3.26	2.49	0.09
Food production	0.30	1.00	0.10	0.10	0.01
Raw material	0.05	0.10	2.60	0.01	0.05
Entertainment and leisure	0.04	0.01	1.28	4.34	0.11
Total	7.24	6.91	21.85	46.01	0.64

**Table 2:** Annual ESV of each land use type per hectare in Hangzhou (US$·hm^−1^ a^−1^).

Ecosystem Function	Land Use Type				
	Grassland	Cultivated Land	Forest	Water Area	Construction Land
Gas regulation	227.99	142.49	997.45	0.00	0.00
Climate regulation	256.49	253.64	769.46	131.09	0.00
Water conservation	227.99	170.99	911.96	5808.03	8.55
Soil formation and protection	555.72	416.08	1111.45	2.85	5.70
Waste treatment	373.33	467.38	373.33	5181.06	2.85
Biodiversity conservation	310.64	202.34	929.06	709.62	96.90
Food production	85.50	284.99	28.50	28.50	2.85
Raw material	14.25	28.50	740.97	2.85	0.00
Entertainment and leisure	11.40	2.85	364.78	1236.84	2.85
Total	2063.31	1969.26	6226.97	13,112.25	119.69

**Table 3:** Areas of different land use types in ecologically sensitive area of Hangzhou.

Land Use Type	Land Area in Different Years (km^2^)								Change Range (km^2^)	
	2000	%	2005	%	2010	%	2016	%	2000–2010	2011–2016
Grassland	0.95	0.31	0.82	0.27	1.03	0.34	0.49	0.16	0.08	−0.54
Urban land	7.67	2.51	17.18	5.62	22.02	7.21	65.35	21.39	14.35	43.33
Cultivated land	182.41	59.71	172.42	56.44	160.4	52.51	153.48	50.24	−22.01	−6.92
Forest	90.24	29.54	92.01	30.12	85.53	28.00	59.43	19.46	−4.71	−26.1
Rural settlements	10.58	3.46	8.68	2.84	19.19	6.28	12.24	4.01	8.61	−6.95
Water area	13.28	4.35%	14.15	4.63%	17.15	5.61%	14.36	4.70	3.87	−2.79
Unused land	0.35	0.11%	0.22	0.07%	0.17	0.06%	0.12	0.04	−0.18	−0.05

**Table 4:** Total ESV based on land use in ecologically sensitive area of Hangzhou.

Ecosystem Function	Unit Value (US$·hm^−2^ a^−1^)	Total ESV (million $)			
		ESV_2000_	ESV_2005_	ESV_2010_	ESV_2016_
Grassland	2063.31	0.20	0.17	0.21	0.10
Cultivated land	1969.26	35.92	33.95	31.59	30.22
Forest	6226.97	56.19	57.29	53.26	37.01
Water area	13,112.25	17.41	18.55	22.49	18.83
Unused land	119.69	0.00	0.00	0.00	0.00
Rural settlements	119.69	0.13	0.10	0.23	0.15
Urban land	119.69	0.09	0.21	0.26	0.78
Total	23,730.86	109.95	110.28	108.04	87.09

**Table 5:** Total ESV in different towns in ecologically sensitive area of Hangzhou.

	Average ESV								Total ESV (US$ million)				Change Rate
	2000	Order	2005	Order	2010	Order	2016	Order	2000	2005	2010	2016	
Banqiao Town	0.42	4	0.44	4	0.45	3	0.39	2	2.14	2.22	2.26	1.99	−7.00%
Cangqian Town	0.12	10	0.11	10	0.11	10	0.09	10	10.23	9.89	9.49	7.69	−24.87%
Hengfan Town	0.46	3	0.47	3	0.44	4	0.31	4	8.52	8.76	8.27	5.83	−31.49%
Jincheng Town	0.46	2	0.48	2	0.49	2	0.38	3	16.19	16.98	17.11	13.46	−16.86%
Liangzhu Town	0.18	9	0.19	9	0.17	9	0.17	7	1.23	1.26	1.17	1.16	−5.35%
Qingshanhu Town	0.52	1	0.54	1	0.54	1	0.44	1	29.95	30.99	31.11	25.05	−16.38%
Wuchang Town	0.33	5	0.27	7	0.24	7	0.11	9	6.26	5.21	4.57	2.02	−67.71%
Xianlin Town	0.25	8	0.25	8	0.22	8	0.15	8	4.47	4.34	3.93	2.58	−42.17%
Yuhang Town	0.32	6	0.32	5	0.31	5	0.29	5	27.98	27.76	27.3	25.46	−9.00%
Zhongtai Town	0.29	7	0.28	6	0.27	6	0.18	6	2.98	2.87	2.81	1.85	−37.98%
Total	0.32		0.32		0.31		0.25		109.95	110.28	108.04	87.09	−20.79%

**Table 6:** Transition matrix of land use and ESV between 2000 and 2016 in the western suburban area of Hangzhou.

	Final State (2016)							
Initial State (2000)	Grassland	Urban Land	Cultivated Land	Forest	Rural Settlements	Water arEa	Unused Land	Total (2000)
Land Use Transfer (km^2^)								
Grassland	0.13	0.07	0.44	0.26	0.04	0.00	0.00	0.95
Urban land	0.00	4.60	2.35	0.13	0.47	0.12	0.00	7.67
Cultivated land	0.05	46.12	112.42	9.03	8.68	6.11	0.00	182.41
Forest	0.17	8.87	30.28	48.90	1.37	0.61	0.05	90.24
Rural settlements	0.02	3.57	5.11	0.49	1.30	0.09	0.00	10.58
Water area	0.01	2.12	2.79	0.56	0.37	7.44	0.00	13.28
Unused land	0.12	0.00	0.10	0.06	0.00	0.00	0.07	0.35
Total 2016	0.49	65.35	153.49	59.43	12.24	14.36	0.12	305.48
ESV transfer (US$ million)								
Grassland	0.00	−0.0136	−0.0041	0.1083	−0.0078	0.00	0.00	0.0827
Urban land	0.00	0.00	0.4346	0.0794	0.00	0.1326	0.00	0.6466
Cultivated land	0.0005	−8.5302	0.00	3.8447	−1.6054	6.8084	0.0000	0.5179
Forest	−0.0708	−5.4172	−12.8923	0.00	−0.8367	0.4200	−0.0305	−18.8275
Rural settlements	0.0039	0.00	0.9451	0.2993	0.00	0.1169	0.00	1.3652
Water area	−0.0110	−2.7544	−3.1089	−0.3856	−0.4807	0.00	0.00	−6.7407
Unused land	0.0233	0.00	0.0185	0.0366	0.00	0.00	0.00	0.0785
Total	−0.0541	−16.7154	−14.6071	3.9827	−2.9306	7.4779	−0.0305	−22.8773

**Table 7:** Elasticity of ESV with respect to land use/cover change (LUCC) in ecologically sensitive area of Hangzhou.

Town Name	Elasticity of ESV in Relation to LUCC		
	2000–2005	2006–2010	2011–2016
Banqiao Town	1.79	0.42	3.95
Cangqian Town	2.88	1.30	3.78
Hengfan Town	1.87	2.27	3.94
Jincheng Town	2.16	0.34	3.32
Liangzhu Town	2.71	2.19	0.81
Qingshanhu Town	1.41	0.13	3.08
Wuchang Town	2.23	3.41	2.90
Xianlin Town	0.48	4.40	3.50
Yuhang Town	0.92	1.03	3.41
Zhongtai Town	1.09	0.48	8.63
Total	0.19	0.84	4.08

## References

[1] Ye Y., Bryan B.A., Connor D., Chen L., Qin Z., He M. (2018). Changes in land-use and ecosystem services in the Guangzhou-Foshan Metropolitan Area, China from 1990 to 2010: Implications for sustainability under rapid urbanization. Ecol. Indic..

[2] Polasky S., Nelson E., Pennington D., Johnson K.A. (2011). The Impact of Land-Use Change on Ecosystem Services, Biodiversity and Returns to Landowners: A Case Study in the State of Minnesota. Environ. Resour. Econ..

[3] Wang X., Dong X., Liu H., Wei H., Fan W., Lu N., Xu Z., Ren J., Xing K. (2017). Linking land use change, ecosystem services and human well-being: A case study of the Manas River Basin of Xinjiang, China. Ecosyst. Serv..

[4] Li Y., Zhan J., Liu Y., Zhang F., Zhang M. (2018). Resources, Conservation and Recycling Response of ecosystem services to land use and cover change: A case study in Chengdu City. Resour. Conserv. Recycl..

[5] Wu Y., Shan L., Guo Z., Peng Y. (2017). Cultivated land protection policies in China facing 2030: Dynamic balance system versus basic farmland zoning. Habitat Int..

[6] Tao Y., Wang H., Ou W., Guo J. (2018). A land-cover-based approach to assessing ecosystem services supply and demand dynamics in the rapidly urbanizing Yangtze River Delta region. Land Use Policy.

[7] Song W., Deng X. (2017). Land-use/land-cover change and ecosystem service provision in China. Sci. Total Environ..

[8] Olushola A., Deng X., Abiodun O., Elijah A. (2018). Science of the Total Environment Assessing changes in the value of ecosystem services in response to land-use/land-cover dynamics in Nigeria. Sci. Total Environ..

[9] Ives C.D., Kendal D. (2013). Land Use Policy Values and attitudes of the urban public towards peri-urban agricultural land. Land Use Policy.

[10] Lee Y.C., Ahern J., Yeh C.T. (2015). Ecosystem services in peri-urban landscapes: The effects of agricultural landscape change on ecosystem services in Taiwan’s western coastal plain. Landsc. Urban Plan..

[11] Tian L., Zhao M., Peng J., Hu Y., Wu J., Liu Y. (2017). Ecosystem services response to urbanization in metropolitan areas: Thresholds identification. Sci. Total Environ..

[12] Huang L., Zhang Y., Shi Y., Liu Y., Wang L., Yan N. (2015). Estuarine, Coastal and Shelf Science Comparison of phosphorus fractions and phosphatase activities in coastal wetland soils along vegetation zones of Yancheng National Nature Reserve, China. Estuar. Coast. Shelf Sci..

[13] Hou H., Wang R., Murayama Y. (2019). Scenario-based modelling for urban sustainability focusing on changes in cropland under rapid urbanization: A case study of Hangzhou from 1990 to 2035. Sci. Total Environ..

[14] Lyu R., Zhang J., Xu M., Li J. (2018). Impacts of urbanization on ecosystem services and their temporal relations: A case study in Northern Ningxia, China. Land Use Policy.

[15] Brüll A., van Bohemen H., Costanza R., Mitsch W.J. (2011). Procedia Environmental Sciences Benefits of ecological engineering practices. Procedia Environ. Sci..

[16] Costanza R., de Groot R., Sutton P., van der Ploeg S., Anderson S.J., Kubiszewski I., Farber S., Turner R.K. (2014). Changes in the global value of ecosystem services. Glob. Environ. Chang..

[17] Li Y., Feng Y., Guo X., Peng F. (2017). Changes in coastal city ecosystem service values based on land use—A case study of Yingkou, China. Land Use Policy.

[18] Wan L., Lee J., Ye X., Zheng L., Wu K., Lu X. (2014). Effects of urbanization on ecosystem service values in a mineral resource-based city. Habitat Int..

[19] Xinzhang S., Hailin Z., Gaodi X. (2007). Ecological Functions and Their Values in Chinese Cropland Ecosystem. China Popul. Res. Environ..

[20] Zhang B., Li W., Xie G. (2010). Ecosystem services research in China: Progress and perspective. Ecol. Econ..

[21] Fu B., Li Y., Wang Y., Zhang B., Yin S., Zhu H., Xing Z. (2016). Evaluation of ecosystem service value of riparian zone using land use data from 1986 to 2012. Ecol. Indic..

[22] Liu S., Costanza R. (2010). Ecosystem services valuation in China. Ecol. Econ..

[23] Gashaw T., Tulu T., Argaw M., Worqlul A.W., Tolessa T. (2018). Estimating the impacts of land use/land cover changes on Ecosystem Service Values: The case of the Andassa watershed in the Upper Blue Nile basin of Ethiopia. Ecosyst. Serv..

[24] Leh M.D.K., Matlock M.D., Cummings E.C., Nalley L.L. (2013). Quantifying and mapping multiple ecosystem services change in West Africa. Agric. Ecosyst. Environ..

[25] Wu K., Ye X., Qi Z., Zhang H. (2013). Impacts of land use/land cover change and socioeconomic development on regional ecosystem services: The case of fast-growing Hangzhou metropolitan area, China. Cities.

[26] Costanza R., Arge R., DeGroot R., Farberk S., Grasso M., Hannon B., Limburg K., Naeem S., Neill R.V.O., Paruelo J. (1997). The value of the world’s ecosystem services and natural capital. Nature.

[27] Li B., Chen D., Wu S., Zhou S., Wang T., Chen H. (2016). Spatio-temporal assessment of urbanization impacts on ecosystem services: Case study of Nanjing City, China. Ecol. Indic..

[28] Zhou D., Tian Y., Jiang G. (2018). Spatio-temporal investigation of the interactive relationship between urbanization and ecosystem services: Case study of the Jingjinji urban agglomeration, China. Ecol. Indic..

[29] Chuai X., Huang X., Wu C., Li J., Lu Q., Qi X., Zhang M., Zuo T., Lu J. (2016). Land use and ecosystems services value changes and ecological land management in coastal Jiangsu, China. Habitat Int..

[30] Englund O., Berndes G., Cederberg C. (2017). How to analyse ecosystem services in landscapes—A systematic review. Ecol. Indic..

[31] Xue M., Luo Y. (2015). Dynamic variations in ecosystem service value and sustainability of urban system: A case study for Tianjin city, China. Cities.

[32] Barbier E.B. (2015). Valuing the storm protection service of estuarine and coastal ecosystems. Ecosyst. Serv..

[33] Long H., Liu Y., Hou X., Li T., Li Y. (2014). Effects of land use transitions due to rapid urbanization on ecosystem services: Implications for urban planning in the new developing area of China. Habitat Int..

[34] Zhu Y., Reid B.J., Meharg A.A., Banwart S.A., Fu B. (2017). Science of the Total Environment Optimizing Peri-URban Ecosystems (PURE) to re-couple urban-rural symbiosis. Sci. Total Environ..

[35] Gonc J., Castilho M., Ezequiel S., Moreira F., Loupa-ramos I. (2017). Land Use Policy Differentiating peri-urban areas: A transdisciplinary approach towards a typology. Land Use Policy.

[36] Li F., Ye Y.P., Song B.W., Wang R.S., Tao Y. (2014). Assessing the changes in land use and ecosystem services in Changzhou municipality, Peoples’ Republic of China, 1991–2006. Ecol. Indic..

[37] Wang J., Zhou W., Pickett S.T.A., Yu W., Li W. (2019). A multiscale analysis of urbanization effects on ecosystem services supply in an urban megaregion. Sci. Total Environ..

[38] Zhang D., Huang Q., He C., Wu J. (2017). Impacts of urban expansion on ecosystem services in the Beijing-Tianjin-Hebei urban agglomeration, China: A scenario analysis based on the Shared Socioeconomic Pathways. Resour. Conserv. Recycl..

[39] Fei L., Shuwen Z., Jiuchun Y., Liping C., Haijuan Y., Kun B. (2018). Effects of land use change on ecosystem services value in West Jilin since the reform and opening of China. Ecosyst. Serv..

[40] Du X., Huang Z. (2017). Ecological and environmental effects of land use change in rapid urbanization: The case of hangzhou, China. Ecol. Indic..

[41] Zhong T., Huang X., Zhang X., Scott S., Wang K. (2012). The effects of basic arable land protection planning in Fuyang County, Zhejiang. Appl. Geogr..

[42] Xu G., Huang X., Zhong T., Chen Y., Wu C., Jin Y. (2015). Assessment on the effect of city arable land protection under the implementation of China ’ s National General Land Use Plan (2006 e 2020). Habitat Int..

[43] Yi H., Güneralp B., Filippi A.M., Kreuter U.P., Güneralp İ. (2017). Impacts of Land Change on Ecosystem Services in the San Antonio River Basin, Texas, from 1984 to 2010. Ecol. Econ..

